# Fabrication of Nanocrystalline AlCoCrFeNi High Entropy Alloy through Shock Consolidation and Mechanical Alloying

**DOI:** 10.3390/e21090880

**Published:** 2019-09-10

**Authors:** Ali Arab, Yansong Guo, Qiang Zhou, Pengwan Chen

**Affiliations:** State Key Laboratory of Explosion Science and Technology, Beijing Institute of Technology, Beijing 100081, China

**Keywords:** high entropy alloys, shock consolidation, AlCoCrFeNi, nanocrystalline

## Abstract

High entropy alloys (HEAs) are usually fabricated using arc melting which has the disadvantages of diseconomy, and the limitations in the shape and size of final products. However, recently, quite a large amount of research has been carried out to find the fabrication techniques for HEAs with better properties such as mechanical alloying and rapid solidification. In this paper, an AlCoCrFeNi high entropy alloy was successfully fabricated by the shock consolidation technique. In this method, the starting powders were mixed by mechanical alloying and then the shock wave was imposed to the compacted powders by explosion. High levels of residual stress existed in samples fabricated by the shock consolidation method. Due to this, after fabrication of the sample, heat treatment was used to eliminate the residual stress and improve the mechanical properties. The microstructure of the samples before and after heat treatment were examined by XRD, SEM and electron backscatter diffraction (EBSD). The shock consolidated sample and sample with heat treatment both showed the nano-structure. After heat treatment the hardness of the sample was decreased from 715 HV to the 624 HV, however the failure strength increased, and as expected the ductility of the sample was improved after heat treatment.

## 1. Introduction

Usually alloys have been developed according to the base element model. This strategy begins with one and rarely two principal elements. Over the last decade, a new class of the alloy system known as high entropy alloys (HEAs) was introduced and developed [1]. HEAs are defined as concentrated solid solutions, containing equiatomic or non-equiatomic quantities of five or more principal elements [2]. The HEAs present excellent oxidation and corrosion fatigue and wear resistance, high hardness and strength with reasonably good ductility. Numerous HEAs have been synthesized and characterized over the past twenty years [3]. Although HEAs are still widely under an initial state of investigation, obtained experimental results of these alloys have presented excellent specific strength, superior mechanical performance at high temperatures, exceptional ductility and fracture toughness at cryogenic temperatures, and superconductivity. Due to their considerable structural, functional potential as well as the richness of design, HEAs are promising candidates for many applications.

Most of the HEAs have been prepared by the arc melting and casting route. This method has disadvantages of diseconomy and limitations in the shape and size of final products [4]. However, recently a large amount of research has been done to fabricate nanocrystalline HEAs [5,6,7]. It is well accepted that the nanocrystalline (smaller than 500 nm) material shows better mechanical properties compared with their coarse-grained counterparts. This enhancement is due to the large fraction of grain boundaries which plays important roles in the plastic deformation and fracture of nanocrystalline alloys. Mechanical alloying (MA) is one of the most effective way to produce nanocrystalline HEAs powder. Mechanical alloying processing inevitably requires additional steps of consolidation such as spark plasma sintering, hot isostatic pressing or powder sintering, and this may introduce undesirable grain growth [1,8,9]. Varalakshmi et al. [10] synthesize nanocrystalline equiatomic CuNiCoZnAlTi HEAs powder by mechanical alloying and they find that as-milled HEAs powder is composed of Body-centered cubic (BCC) phase with crystallite size less than 10 nm. They consolidate the powder using vacuum hot press (at 800 °C) and achieve high hardness (7.55 GPa) and compressive strength (2.3 GPa). However, they observe grain growth during the sintering process, and find the grain size of the sample after the sintering is increased to 59 nm. Wang et al. [11] synthesize the BCC phase CoCrFeNiMnAl powder by ball milling and consolidate powder by spark plasma. They observe that BCC phase and Face-centered cubic (FCC) phase coexist in the consolidated sample. Mohanty et al. [12] synthesize AlCoCrFeNi by ball milling and consolidate by spark plasma sintering. Youssef et al. [13]. fabricate nanocrystalline Al_20_Li_20_Mg_10_Sc_20_Ti_30_ HEA powder by mechanical alloying, and they observe the phase change (FCC to hexagonal close-packed (HCP)) after annealing. However, they achieve super-low density (3 gr/cm^3^) and high hardness (6.1 GPa) by this method. Yim et al. [14] use mechanical alloying to synthesize the CoCrFeMnNi HEA powder. Then, they use a gas gun to generate the shock wave to compact the powders instead of a static compaction method. Compacted powders sre sintered by pressure-less sintering. With this method, they achieve a high relative density (99.5%); however, they cannot achieve nanocrystalline HEA by this method (the final grain size is 4.08 μm). Nearly instantaneous heating of powder is required for the retention of nanocrystallinity, which limits the application of most conventional fabrication methods. Other methods such as severe plastic deformation also have its limitation in the size of samples.

Rapid solidification technology is a rapidly advancing field, and unique microstructures of metallic alloys and ceramics have been created [15,16,17,18,19]. Shock consolidation (SC) is a unique technique, using a shock wave generated by explosion or impact, shows considerable promise for producing bulk material from powders [19]. As the shock wave propagates the powders, the particles are compressed to an ultra-high pressure (> 1 GPa) that is far higher than the yield strength of the particles, leading to severe deformation, and a high temperature exceeding the melting point is achieved instantaneously due to the shock energy deposition through plastic deformation, friction and adiabatic compression. Due to the rapid cooling rate of 10^9^ K/s, the high temperature is limited to the surface while the interior of particle remains relatively cool. These inherent properties make the shock consolidation a promising method to fabricate nanocrystalline materials. Emelchenko et al. [20] report that fully dense nanocrystalline Ni bulk is successfully obtained using shock consolidation, with the averaged grain size of 56 nm identical to the initial size. Nanocrystalline NiAl [21], copper [22], aluminum [23,24] etc. are produced by this method. This research reveals that nanocrystalline materials can be produced by SC. However, no research has been done on the fabrication of the nanocrystalline HEAs by SC.

Al*_x_*CoCrFeNi has great mechanical properties. This class of HEAs exhibits a range of microstructures and properties [25]. The microstructure of Al*_x_*CoCrFeNi can be tailored from single FCC solid solution, duplex FCC plus BCC solid solutions to single BCC solid solution by increasing the aluminum mole ratio, meanwhile the hardness increases from 116 HV to 509 HV. Al*_x_*CoCrFeNi is fabricated by different methods such as arc melting, microwave sintering [3,26,27,28].

In this research, the nanocrystalline AlCoCrFeNi powders fabricated via ball milling was consolidated to fully dense bulk without any cracks by shock wave. The following heat treatment (HT) was conducted to eliminate the residual stress and improve the bonding strength. The microstructures of the fabricated sample were studied via X-ray diffraction (XRD), Energy-dispersive X-ray spectroscopy (EDS), Scanning Electron Microscopy (SEM) and electron backscatter diffraction (EBSD), and the mechanical properties were also investigated by hardness measurement and quasi-static compressive test.

## 2. Materials and Methods

**Figure 1:**
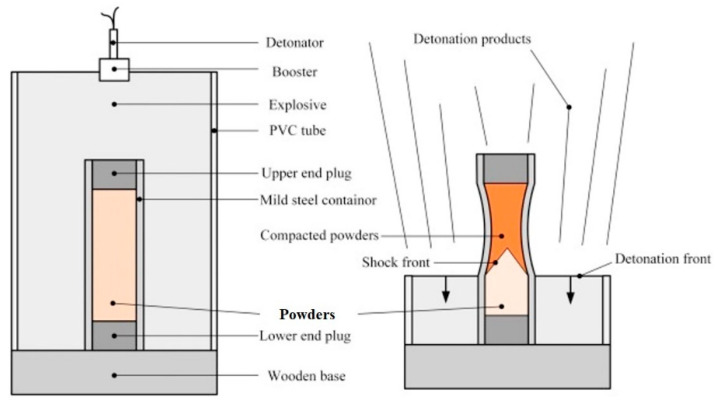
Schematic of the setup and process of shock consolidation technique.

High purity elemental (99.5%) powders (average particle sizes of 45 μm) of Al, Co, Cr, Fe and Ni with equiatomic compositions were used as starting materials. The elemental powders were milled in a planetary ball-miller for 60 h at 300 rpm in an argon atmosphere. High-performance stainless steel vials and balls were utilized as the milling media with a ball-to-powder mass ratio of 10:1. To prevent overheating the milling process was stopped every 1 h for 10 min. The shock consolidation experiments were carried out using a setup with cylindrical configuration, as shown in Figure 1. The steel tube was filled with milled powders (MA powders) which was uniaxial pressed to reach a 70% of theoretical density, and sealed by steel caps at both ends; then the steel tube was surrounded by powder explosive named expanded ammonium nitrate with a detonation velocity of 2300 m/s. A detonator was used to ignite the powder explosive at the top; and a cone-shaped shock wave was formed in the HEA powders as the detonation wave traveled inward and propagated from top to bottom with the detonation, as shown by Figure 1. Uniform distribution of high pressure could be achieved within the cross-section of HEA powders by adjusting the thickness of the explosive. After explosion, the compacted tubes were extracted out and cut into halves. One piece was investigated as the SC sample and the other (HT sample) was heat treated at a temperature of 800 ºC for 120 h. Due to the slow diffusion processes in HEAs (so-called sluggish diffusion), HEAs need prolonged annealing time for any heat treatment process compared to other metals and alloys [29,30,31].

Sample surfaces were ground using silicon carbide papers (300, 600, 1000 and 1500), after this step the sample surfaces were mechanically polished using the Fe_2_O_3_ and Cr_2_O_3_ powders and finally etched with a solution consisting of 80wt% distilled water, 15wt% HNO_3_ and 5wt% HF for microstructural observation. For phase identification, an X-ray diffractometer (XRD) (Bruker AXS D2 Advance, Billerica, MA, USA) was utilized. Scanning electron microscopy (SEM) was carried out on a Hitachi S-4800 (Hitachi, Tokyo, Japan). Samples for electron backscatter diffraction (EBSD) (Quanta, OR, USA) samples were prepared by conventional metallographic polishing followed by electro-polishing using a 5% perchloric acid and 95% glacial acetic acid (by volume) mixture at 303 K.

The Vickers hardness was measured with a load of 100 g and dwelling time of 15 s. A total of 30 tests were carried out on three different specimens. The average values of these measurements were reported as the Vickers hardness of the sample. Quasi static compression was conducted on cylindrical specimens (diameter of 3.5 mm and length 5.25 mm) by using the Instron and three samples were tested at a strain rate of 10^−3^ s^−1^. The top and bottom faces of the specimens were cleaned by the SiC paper to ensure all faces were flat. The density of the sample was measured by the Archimedes method. The density of five different specimens was measured and the average of these readings was reported as the density of the sample. The theoretical density of the sintered specimens was calculated by applying the rule of mixture, using theoretical densities of the individual elements. The relative density of the samples was calculated by taking the ratio of measured and theoretical densities.

## 3. Results

**Figure 2:**
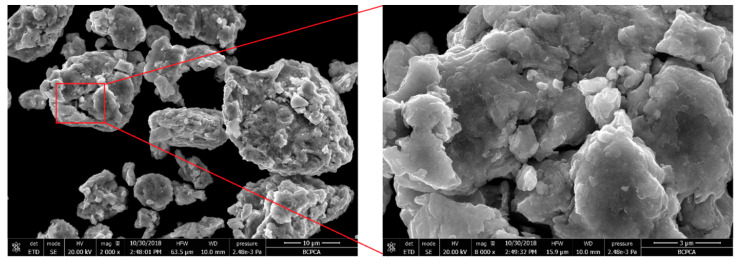
SEM image of the AlCoCrFeNi powders after milling.

**Figure 3:**
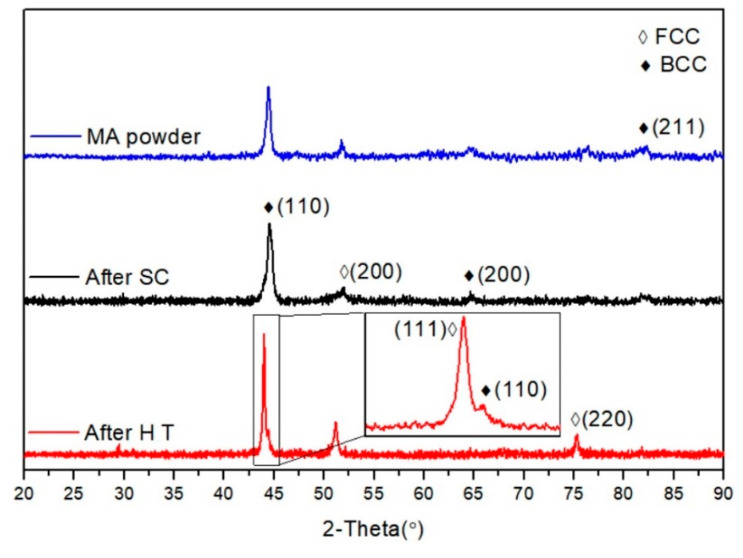
XRD patterns of the mechanical alloying (MA) powders and the high entropy alloy (HEA) after shock consolidation (SC) and heat treatment (HT).

Figure 2 shows the SEM micrograph of the mixed powder (Al, Co, Cr, Fe and Ni) after 60 h milling. The image shows the HEA particles had a predominantly irregular shape the heavy fracture observed in the particles. During the ball milling process, continuous fracturing, deformation as well as cold welding increases the diffusivity of the starting elements and lead to the formation of the HEA [28]. The XRD pattern of the ball milled powders, shock consolidated sample and shock consolidation with heat treatment are shown in the Figure 3. No peaks of pure elements were detected and BCC and FCC structures could be identified in the MA powders and SC sample; it indicated that the starting elements (Al, Co, Cr, Fe and Ni) are distributed in the crystal lattices [32] and the solid solution of HEA was successfully fabricated through ball milling. Wang et al. [33] fabricated Al*_x_*CoCrFeNi by arc melting and find that if the amount of x is higher than 0.9 only BCC diffraction peaks are identified. Zhang et al. [26] find the FCC, BCC and B2 in the AlCoCrFeNi sample sinter by spark plasma. Shivam et al. [28] report a similar pattern for the ball milled powders. They find a solid solution phase is formed after 20 h milling, and with further milling, a more homogenized solid solution with finer grains can be achieved. The XRD pattern of milled powders showed lowering and broadening of peaks and its subsequent disappearance may have resulted from the following three factors: grain refinement, high lattice strain and decreased crystallinity [34]. The grain size was estimated from the XRD pattern by using the Scherrer equation. The averaged grain size of the MA powders was 26 nm, it indicates that the micro-scaled powders shown in Figure 2 were actually the agglomerations of nano-sized grains. Due to the metastable nature of HEAs, the phase composition changed as long as the synthetic condition varied.

The main phases of the MA powders and the as-received sample showed BCC structure; after HT, the FCC structure appeared clearly and became the dominated structure. Peaks belonging to the BCC structure had nearly disappeared, but peaks of (110) was still present. The (200) FCC peak was shifted to lower angle after HT, which was consistent with the results of Wang et al. [32].

**Figure 4:**
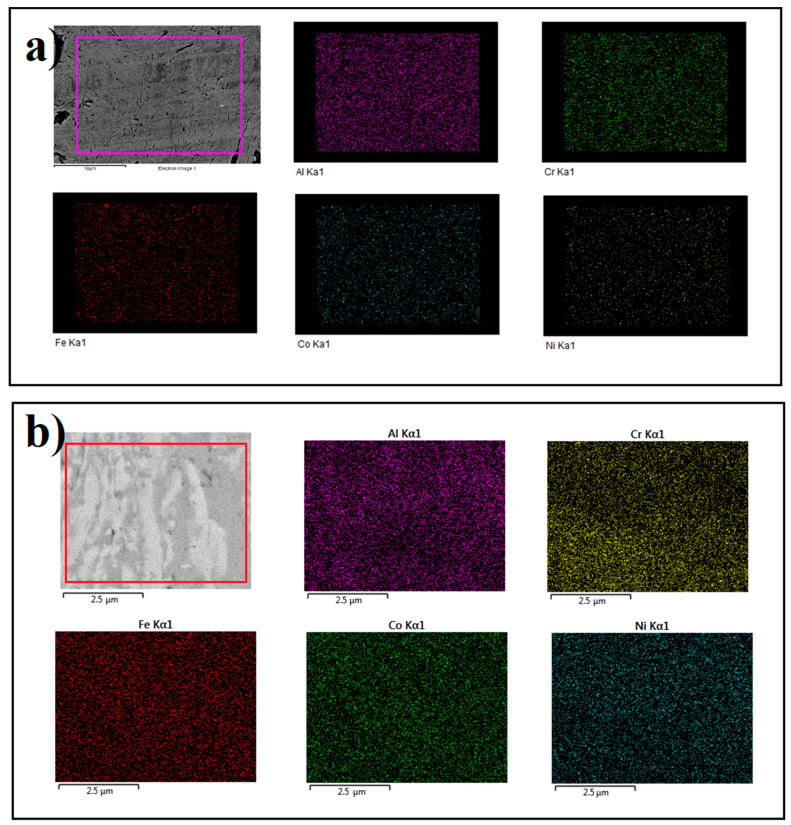
SEM and energy dispersive X-ray spectroscopy (EDX) mapping of the (**a**) shock consolidate AlCoCrFeNi and (**b**) heat treatment shock consolidate AlCoCrFeNi.

**Figure 5:**
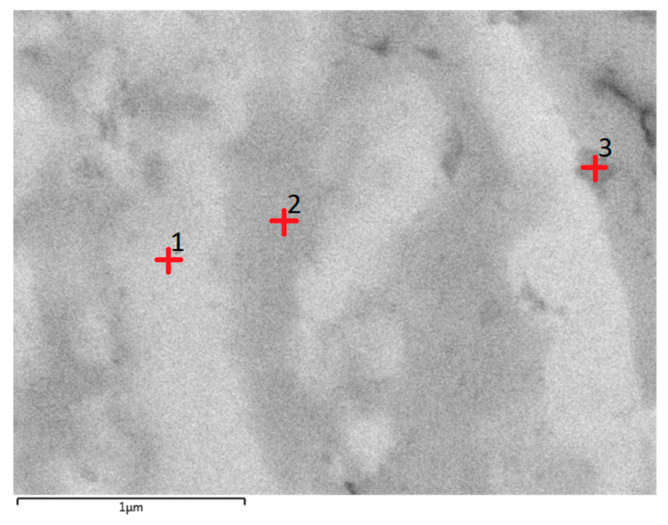
The position of three specific points for elemental analysis of shock consolidated HEA after heat-treatment.

The densities of both SC and HT samples reached 7.1 g/cm^3^, nearly identical to the theoretical density of AlCoCrFeNi HEA and were higher than the AlCoCrFeNi HEAs fabricated by arc melting, arc plasma sintering or other powder metallurgical methods [12,26]. Chemical compositions of the samples after SC and HT were examined by the energy dispersive X-ray spectroscopy (EDX) (Hitachi, Tokyo, Japan). As shown in Figure 4, all samples showed a quite homogeneous distribution of five elements. Three zones were observed in the microstructure of the HT sample, as indicated by 1, 2 and 3 in Figure 5, and the spectroscopy data are given in Table 1. It shows the heat treatment process led to the separation of some precipitates from AlCoCrFeNi. The white zone (point 1) in the SEM image shows almost equiatomic composition with minor variation from perfect stoichiometry. The gray (point 2) and dark zone (point 3) were Fe, Cr-rich phases, corresponding to an FCC phase [16]. Nevertheless, the XRD result showed that a BCC phase still existed in the FCC-dominant HT sample. The white phase may be interpreted as a uniform mixture of BCC and FCC phases.

However, these five elements (Fe, Co, Ni, Al, Cr) are close to each other in the periodic table and due to this a huge difference in the X-ray generation capability is not expected. Therefore, the variation in the chemistry of the particles could be attributed to the diffusion-assisted change in the chemistry of the particles during the heat treatment process. Due to the slow diffusion processes in HEAs (so-called sluggish diffusion), HEAs need prolonged exposure time for any heat treatment process compared to other metals and alloys [29]. Uporove et al. [29] find that to achieve thermodynamically equilibrium state in HEAs, the exposure times should be more than 100 h. Several research studies have been carried out to understand the heat treatment process of the HEAs and effect of the different heat treatment condition on the mechanical properties [29,35,36], however, the details of the heat treatment process of the HEAs is not fully understood yet, and this process is more complicated with the nano-grained initial structure. Tang et al. [37] found reduction in grain size to the nanometer scale significantly affects its heat treatment-induced phase transformation pathway, it may be due to the difference in elemental diffusivity in coarse grain and nano-structure materials. Nano structure HEAs can provide the pathway for rapid element diffusion.

**Figure 6:**
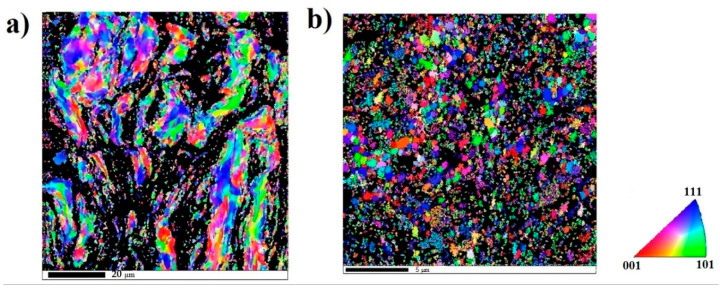
Electron backscatter diffraction (EBSD) image of (**a**) the shock consolidate AlCoCrFeNi and (**b**) heat treated shock consolidated AlCoCrFeNi.

**Figure 7:**
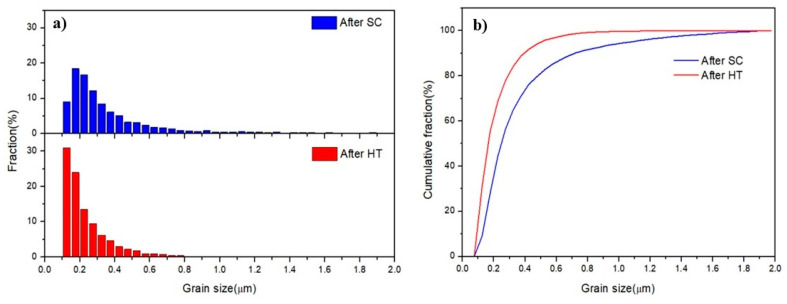
(**a**) Grain size distributions of the SC and HT samples, and (**b**) cumulative percentage of the grain size of the SC and HT samples.

The EBSD analysis of the SC and HT samples are shown in Figure 6. In the EBSD image the dark region was observed because of the large localized plastic deformation and high residual stress that were imposed to the sample during the shock consolidation process. Figure 6a shows the EBSD analysis of the shock consolidated sample. Two kinds of grains were observed in the SC sample, elongated grains and nano-size grains; after HT, the elongated grains disappeared and only equiaxed nanocrystalline grains were observed. Figure 7a presents the grain size distributions of the SC and HT samples, showing that the range of grain size of SC sample varied from 100 nm to 2 μm, and for the HT sample it varied from 100 nm to 1 μm. Figure 7b presents the cumulative percentage of the grain size, showing that 50% grains of SC and HT samples were below 250 nm and 160 nm, respectively. It also shows 80% grains of SC samples and 95% grains of HT samples are below 500 nm, then the grain refinement induced by HT could be concluded from Figure 7b. The refinement may be due to the dissolution of the majority of the existing phases and the creation of very small nano-precipitates [35]. However, in EBSD analysis a huge area was not indexed (showed by the black color), the size of the grain in this area might be less than 50 nm. In addition, it is observed that the black area in the SC sample is higher than the HT sample, and due to this we could not conclude with certainty the heat treatment of the SC sample led to the smaller grain size, but the EBSD image shows that after the heat treatment the microstructure of sample was more homogenous. The mixing enthalpy between Ni–Al is higher than other pairs, which means Al and Ni tend to form atomic pairs and segregate [38] during HT. The precipitation of Ni and Al leads to the formation of Fe–Cr rich FCC phase in the HT sample, which was also confirmed by XRD analysis.

The hardness of the SC sample was measured as 715 HV, which was higher than the AlCoCrFeNi sample fabricated by the arc plasma sintering (518 HV) as well as the values reported in the other literature [12,33,39,40]. The hardness of sample after heat treatment decreased to 624 HV but still was higher than most of conventional alloys. The decrease in hardness after heat treatment was attributed to the phase transition from BCC to FCC. In general, the hardness of a BCC structure is higher than the one of an FCC structure. As shown by Figure 3, the FCC structure was the dominant structure in the HT sample. However, the hardness of the HT sample was dramatically higher than the hardness of 120 HV of the reported coarse-grained FCC structured HEAs [33]. Fu et al. [6] also reports a hardness of 454 HV for nanocrystalline FCC HEAs. The main reason is the nanocrystalline structure, which is consistent with Hall–Petch theory. According to the dislocation theory, the grain boundary hinders the dislocation movement. Therefore, higher loads are required for the dislocation movement and grain deformation for the nanocrystalline, leading to the increase in hardness. In addition, high-density dislocations induced by shock loading also helps improve the hardness.

**Figure 8:**
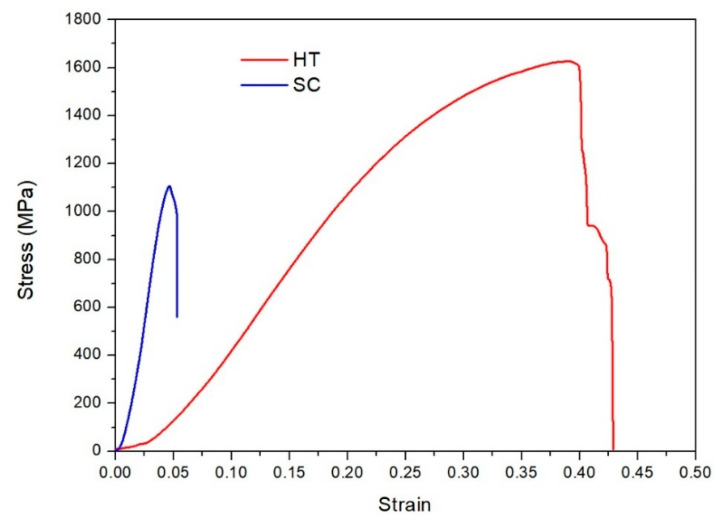
Stress–strain curve of the AlCoCrFeNi.

Figure 8 shows the compressive stress–strain curve of the SC sample and the HT sample at a strain rate of 10^−3^s^−1^. The failure strength of the SC sample was 1.04 GPa, higher than most conventional alloys. However, the SC sample showed a brittle behavior with a failure strain of 5%, which is quite common for the shock consolidated materials due to the high defect density and high residual stress. After HT, both of strength and ductility of the sample were improved, the failure strength increased to 1.63 GPa and the failure strain to 40%. Tang et al. [41] also observe the ductility of the as-cast AlCoCrFeNi HEAs increased after heat treatment. 

**Figure 9:**
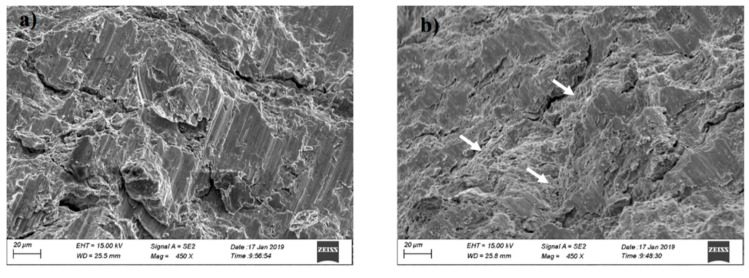
SEM images of the fracture surfaces of (**a**) AlCoCrFeNi and (**b**) AlCoCrFeNi with heat treatment.

Fractographs analysis was carried out using SEM to develop a better understanding of the failure and mechanical behavior of the fabricated samples. All samples showed shear failure with an angle of 45° to the loading axis. Figure 9 shows the fracture surface of the SC sample and HT sample. Typical cleavage fracture could be identified in the SC sample, showing low ductility. Cleavage or quasi-cleavage features are more likely observed on the fracture surface of BCC HEAs [42]. The HT sample with FCC structure shows some small-scale dimple-like zones, as indicated by the arrows in Figure 9b, manifesting better ductility.

In this work, the improvement of ductility is attributed to the phase transition from BCC to FCC. Usually FCC structure HEAs are softer than their BCC counterparts. It is known that deformation occurs by normal FCC slip on the {111} plane in the 〈110〉 direction [43]. In the FCC HEAs high strength can be achieved by grain refinement and dislocation hardening. Besides this, the grain boundary strengthening could be quite crucial because of the nano-crystalline structure. Fu et al. [6] produce the nanocrystalline Co_25_Ni_25_Fe_25_Al_7.5_Cu_17.5_ HEA by using the MA and spark plasma sintering with FCC structure. They find the compressive strength and hardness of the nanocrystalline HEAs are improved by 834.9% and 251.9%, respectively, compared to the coarse-grain HEA. They find the nanostructure is an excellent way to obtain a good combination of strength and ductility in the HEAs. Niu et al. [44] observe in the nanocrystalline structure Al_0.5_CoCrFeNi when the content of BCC phases decreases by the heat treatment process, the strength of sample increases, and they mention it is due to the nanocrystalline structure which has great significance influence on the properties of sample. The grain boundary strengthening could be calculated using the $\Delta \sigma_{GB}=K^{HP\left(nc\right)}d^{-0.5}$, where *d* is the average grain diameter of the sample and *K^HP(nc)^* is a Hall–Petch coefficient [6]. The Hall–Petch coefficient can be assumed to be around 0.35 MPa·m^−1/2^ for nanocrystalline HEAs. Based on this equation the grain boundary strengthening was around 785 MPa.

The excellent strength of AlCoCrFeNi is also attributed to solid solution strengthening [3] and also related to the concentration of the solute atoms. For HEAs, each atom can be considered as a solute atom. Therefore, it is anticipated that this alloy would show substantial solid solution strengthening [3]. Strain hardening observed in coarse-grain HEAs did not occur in this study, which may be due to the nanocrystalline structure in both SC and HT samples. Dislocations have sufficient space to glide in the coarse-grain, but is limited in the nanocrystalline structure. Fu et al. [6] claim that the dislocation strengthening and grain boundaries strengthening are the dominant strengthening mechanism for nanocrystalline HEAs.

## 4. Conclusions

The nearly full dense AlCoCrFeNi HEA with nanocrystalline was successfully fabricated by mechanical alloying and subsequent shock consolidation of MA powders. The MA powders exhibited a BCC-dominant solid-solution phase mixed with a small amount of FCC phase. After shock consolidation, the bulk HEA showed the same phase composition as the milled powders. The bulk HEA consist of nano-sized grains, with the number fraction of ultra-fine grains (UFGs) < 500 nm reaching 80%. After heat treatment, the bulk HEA exhibited an FCC-dominant phase and the number fraction of UFGs < 500 nm increased to 95%, with 50% grains in a size lower than 160 nm. The phase transition and grain refinement could be contributed to the precipitation of Ni and Al during the heat treatment. The nanocrystalline HEA after SC exhibited a brittle behavior with the compression failure strength of 1.04 GPa and failure strain of 5%, while the corresponding values for the HEA after HT are 1.63 GPa and 40%. In contrast, the hardness of 715 HV for HEA after SC dropped to 624 HV after HT. The improvement of ductility and the decrease in hardness could be attributed to the phase transition from BCC to FCC during the heat treatment; and the increase in strength and the higher hardness of FCC structure HEA than the one of coarse grain were attributed to the grain boundaries strengthening.

## Figures and Tables

**Table 1:** Chemical compositions of the AlCoCrFeNi HEA after HT.

Zone	Fe (at%)	Co (at%)	Ni (at%)	Al (at%)	Cr (at%)
**1**	20.31 ± 0.2	20.61 ±0.18	20.87 ± 0.1	19.35 ± 0.06	19.33 ± 0.04
**2**	24.16 ± 0.1	20.99 ± 0.35	21.06 ± 0.07	16.62 ± 0.03	17.17 ± 0.06
**3**	24.045 ± 0.15	20.99 ± 0.07	16.68 ± 0.04	14.79 ± 0.06	23.47 ± 0.1
